# Advertised Medicines

**Published:** 1875-12

**Authors:** 


### ADVERTISED MEDICINES.

While there are many such medicines that are very bad, and very dangerous, a few are deserving of all confidence, and almost always accomplish the purpose desired. Our prejudices do not extend to condemning what we know to be good, because it is found in "bad company."

Those remedies that answer our purpose, that are safe and reliable, we shall use, under whatever name or form they are found in the market.

There are two that we *always* employ in the treatment of cases to which they are adapted. One is a homœpathic remedy, in the form of little sugar pellets, called "Van Deren's Remedy," for Bowel Complaint of children. We keep a liberal supply of it on hand, and always use it in the treatment of bowel complaints of children, and it never disappoints us.

We have never lost a little patient of the hundreds we have treated with it. It contains no opium, and nothing that can possibly do harm, in large or small doses.

Our other favorite remedy is "Nelaton's Pile Suppository." It is the best not only for *piles*, but for *costiveness*, that we have ever seen, and we always prescribe it with the most satisfactory results.

Both these medicines are prepared by Hall & Ruckel, Wholesale Druggists, No. 218 Greenwich street, New York. They will send a package of either, post paid, on receipt of 50 cents. Physicians and Druggists supplied with the Van Deren Remedy at $1 per ounce, $5 for eight ounces, $8 a pound.

